# Delineation of Aflatoxicosis on Health and Performance of Water Buffalo (*Bubalus bubalis*) and Its Therapeutic and Nutritional Management

**DOI:** 10.3390/toxins17020097

**Published:** 2025-02-18

**Authors:** Rajesh Kumar, Sanjay Kumar, Supriya Chhotaray, Madhu Singh, Rupali Rautela, Avijit Dey

**Affiliations:** ICAR—Central Institute for Research on Buffaloes, Hisar 125001, Haryana, India

**Keywords:** aflatoxicosis, water buffalo, health, therapy, feeds, nutrition

## Abstract

A symptom of reduced feed intake, conception and progressive emaciation was noticed in the Murrah buffalo farm of the institute with tail gangrene in some buffaloes and the sudden death of many animals. Thus, the objective of the study was for the systemic investigation to find out the causative agents and necessary ameliorative measures. The tail lesion includes alopecia, scales, necrosis, oedematous and a painful area. After thorough examination of the signs and symptoms of the disease, it was speculated that the case may be due to the presence of mycotoxins in the feeds offered to the animals. The severely affected buffaloes that died subjected to post-mortem examination demonstrated liver damage, nephritis and haemorrhages in all the vital organs. The analyses of offered feed revealed a high concentration of aflatoxin B1 content in maize, groundnut cake, cottonseed cake and compound feed mixtures. The case was typically diagnosed as the aflatoxicosis in water buffalo and ameliorative measures viz. the withdrawal of contaminated feeds, supplementation of toxin binder and penta-sulphate mixture in the feed taken sustained animal health and production performances. Severely affected animals with tail gangrene were treated with local disinfectants and antibiotics as well as systemic injection with broad-spectrum antibiotics and supportive vitamins and minerals to recover to their previous stage. Therefore, routine check-ups of feeds are of utmost importance to prevent feeding of aflatoxin-contaminated feeds. Systemic efforts viz. therapeutic management with topical medicines, broad-spectrum antibiotics, supportive therapies with vitamins and antioxidants along with replacement of contaminated feeds and inclusion of peta-sulphate mixture, and a toxin binder are effective in the prevention and control of aflatoxicosis in buffaloes.

## 1. Introduction

Global climatic changes affect animal health and production performances directly through susceptibility to various diseases and indirectly by reducing the quality of feed ingredients due to infestation by pests and insects. Climatic variability is triggering the development of moulds in food–feed crops and about one-fourth of the world’s food crops are affected by mycotoxins [1]. Among the mycotoxins, aflatoxins (B_1_ and B_2_) are the major contaminants of feed ingredients in Asian, African and Latin American countries [2] and are responsible for huge economic loss through the increased mortality and poor performances and reproductive capabilities of animals. It is of great importance not only due to animal effects, but also to human health concerns owing to the excretion of its metabolites (mostly aflatoxin M_1_) through milk and milk products and other animal products which are carcinogenic and immunosuppressive [3]. Although it is a global issue, the feeds of developing countries are more susceptible to mould growth due to higher humidity and temperature, as well as poor storage conditions [4,5]. As detection and quantification of aflatoxin is not easy, there is a prodigious chance of possible contamination of aflatoxin in food–feed crops and intoxication in animals and human beings [6].

Milk is one of the most important agricultural commodities worldwide. With the highest rank in India, Asia is the largest milk-producing region in the world [7]. With a population of more than 97% of the world’s buffalo in Asia, India ranks first with about 57% of the total, contributing about half of the total milk production of the nation [8]. Buffalo farming also plays a significant role in food security and poverty alleviation in Asian countries. Feed cost accounts for more the 60% of total animal rearing expenditure; therefore, quality feeds are of utmost importance for enhancing the productivity and health of animals. However, low-quality moulded feeds could drastically affect the production and reproduction of animals resulting in huge economic loss [9].

Aflatoxicosis causes reduced feed intake, emaciation, alopecia, sluggish movement, gastrointestinal dysfunction, reduced conception and even death due to liver damage and immunosuppressive effects [10]. Gangrenes in the extremities are not uncommon due to mycotoxicosis. Tail gangrene in buffaloes and cattle has been reported in various states in India, including Punjab, Haryana, Kerala and Uttar Pradesh [11]. A symptom of reduced feed intake, emaciation and conception was noticed in the buffalo farm of the institute with tail gangrene in some buffaloes and the sudden death of many animals. Therefore, the objective of this study was for the systemic investigation to find out the causative agents and necessary ameliorative measures.

## 2. Results

### 2.1. Symptoms and Post-Mortem Findings

The symptoms evidenced were tail alopecia, oedema, necrosis and necrotic rings surrounding the tail. In some animals, it affected less than 3 inches of the tail, while in some it extended about 12 inches. The category-wise number of animals affected is given in Table 1.

**Figure 1:**
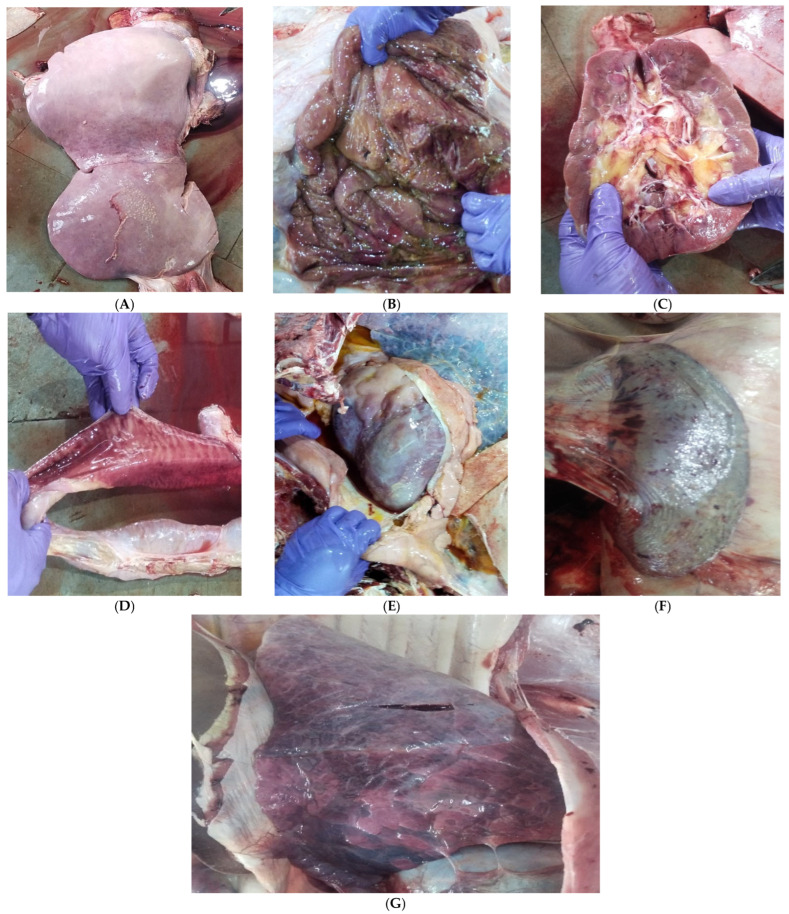
Gross findings of post-mortem of different vital organs: (**A**) haemorrhages and congestion in liver with paleness, (**B**) oedematous abomasum with congestion and ulceration, (**C**) haemorrhages in kidney, (**D**) haemorrhages in intestinal mucosa, (**E**) fluid filled pericardium, (**F**) petechial haemorrhages in the spleen, (**G**) congestion in lungs.

A total of 38 mortalities were reported on the farm with a herd strength of 524 buffaloes between March to September in 2023, and the incidence was highest among 6–12 months old buffalo calves (Table 2). The majority of the vital organs showed signs of haemorrhages and congestion, while cirrhosis was seen in the liver with discolouration (Figure 1). The detailed gross and histological changes in the organs have been presented in Supplementary Table S1.

### 2.2. Report of Feed Analyses

The samples of each feed ingredient that were used for the preparation of concentrate mixture at the time of onset of the outbreak were analysed. It was found that groundnut cakes and cottonseed cakes were severely affected with aflatoxin B-1 with infestation in other ingredients and compound feed mixture also (Table 3).

### 2.3. Biochemical and Haematological Profile

The blood samples from the affected and recovering buffaloes were investigated for the prognosis of the disease and therapeutic management was carried out accordingly for the recovery of the animals. It was observed that PCV% was increased (*p* < 0.05) in many affected buffaloes. Total serum bilirubin was also increased (*p* < 0.05) in the affected individuals. The hyper-bilirubinaemia was also indicative of the haemolysis, thus the increased PCV. Detailed biochemical and haematological profiles of the affected buffaloes has been presented in the Supplementary File (Tables S2 and S3).

### 2.4. Effects on Production and Reproduction Performances in Murrah Buffaloes

The month-wise overall farm animals’ performances were compared with the past two years of average performances with a chi-square goodness-of-fit test. It was observed that the conception rate was significantly affected due to aflatoxin exposure with an observed chi-square value of 51.55, whereas the critical table value at 6 degrees of freedom for α = 0.05 was 12.592. The herd average and wet average did not show any significant difference with a chi-square value of 0.39 and 0.14, respectively. Below, Table 4 highlights the month-wise variation in the performances after mycotoxin exposure, and the effect was mostly observed on the conception rate in the months of the June and July. The effect of aflatoxin in the feed was mostly observed in herd performances after 2 months of the onset of exposure. There was a 91.66% increase in the abortion rate during the mycotoxin outbreak compared to the previous year (2022), out of which 83.33% of abortions were in the second trimester.

### 2.5. Effects of Nutritional and Therapeutic Management

**Figure 2:**
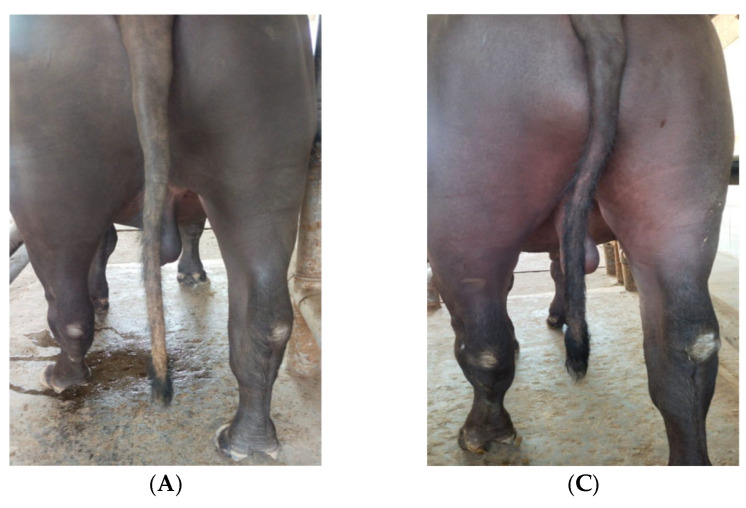

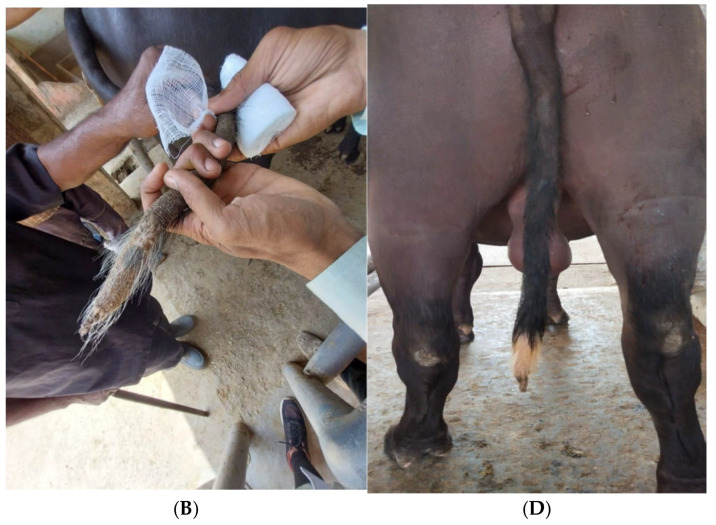
(**A**–**D**) Tail gangrene before (**A**,**B**) and after (**C**,**D**) treatment in affected buffalo bull.

All the affected animals were managed with a combined strategy of feeding of penta-sulphate mixture and systematic and topical treatment of the disease. Systematically, oxytetracycline LA was used to prevent the secondary bacterial infection; Dozliv^®^ has L-carnitine, a hepato-protective agent, and V-5 has vitamins A, D, E and H required for epithelialization of the damaged skin of the tail. Topically, the concentration of the phenol was gradually increasing day by day to evoke a sustained inflammatory response at the site of application. On fourth day of the treatment, an increasing trend of inflammation was noticed. On sixth day, the cold extremity of the tail appeared hot to touch due to increased blood supply as a result of vasodilatation. The scavenger cells started to function by clearing dead cell debris and blood clots. Within 10 days, necrosis localised on the tip of the tail, oedema and pain subsided, scaly growth and oily exudate deposited on the tail disappeared and necrotic rings healed. The regeneration of hair follicles and growth of new hairs on the tail and whole body started from the second week onwards. Generalized health conditions of all the animals started to improve after 15 days of the treatment. The complete recovery of all affected animal tails occurred within a month (Figure 2A–D).

## 3. Discussion

Limited up-to-date aetiological surveys have been performed regarding the tail necrosis cases. It is a matter of debate to date due to a lack of knowledge about the cause of the disease. Some workers claimed that chronic selenosis is responsible for gangrenous syndrome in cattle and buffaloes [12,13]. As a curative, a penta-sulphate mixture is used in case of selenium toxicity because it has antagonistic properties. It has been reported that penta-sulphate mixture has a cure rate of 80% in case of degnala disease as it binds with an excess of selenium in the feed and thus reduces the lethal concentration of the selenium in the feed and excess of selenium absorbed in the body [14].

Other workers considered that gangrenous syndrome is a disease that occurs due to feeding of mycotoxins in the feed above the threshold level. It is produced by Claviceps, Fusarium, Aspergillus and Epichloe [15,16,17,18]. Specifically, degnala disease is believed to be a mycotoxicosis that is characterised by the development of oedema, necrosis and gangrene of the tail, legs and ears in cattle and buffaloes [19,20]. The disease is associated with rice straw feeding and is reported to be induced by a fungal infestation of straw [16,21]. Saprophytic fungi causing the infestation of rice straw produce mycotoxins resulting in vasoconstriction and thrombus formation at the distal extremity especially of the tail; therefore, the lesions of the disease occur [22]. High levels of aflatoxin B1 concentration in the feed ingredients and compound feed mixture of the animals (Table 3) in the present study demonstrated the role of aflatoxicosis in mortality, tail gangrene and reduced feed intake in affected animals [23]. Liver damage, jaundice, congestion and haemorrhages in various vital organs, as described in the classical aflatoxicosis in cattle [24], have also been demonstrated in the present study and supported our findings as a case of aflatoxicosis in buffalo.

Aflatoxins are major contaminants of food crops throughout the world and hot humid conditions of tropical and sub-tropical countries predispose the growth of mycotoxin in both pre-harvest and post-harvest stages of crops [1]. Among the feed ingredients used for animals, maize, wheat, barley and oilseeds, including groundnut, sunflower, soybeans, mustard and cottonseed cakes, are the most susceptible [2]. In the present study, a high concentration of aflatoxin B1 in maize, groundnut cake and cottonseed cake resulted in increased aflatoxin B1 in the compound feed mixture (Table 3) and supports the reduced feed intake, emaciation, digestive problems and death of the animals due to damage of the liver and other vital organs (Figure 1). The root cause of aflatoxin contamination of feed ingredients in the present study could be the storage conditions in the feed store unit of the institute, where some damages on the roof were observed as well as weather circumstances, resulting accumulation of moisture [4]. However, as a source of contaminated feed ingredients might be a vital reason for affecting the total heard of buffaloes, regular feed testing, better storage and selection of better feed suppliers could minimise the risk of aflatoxicosis.

The restriction of blood supply to the distal end of the tail causes hypoxic cell death, and finally necrosis of the distal part of the tail occurs. It requires early attention and proper treatment otherwise thrombi proliferate towards the proximal end and the whole tail becomes gangrenous. In treating gangrene and necrosis of the extremities, vasodilating agents have a very beneficial role, as these agents help in the restoration of the blood flow at the site of application by vasodilatation action and dissolution of the clots or thrombosis formed in the vessels [17]. Phenol of pharma grade is supposed to have vasodilating properties at the site of topical application. It is generally used as a disinfectant, but phenol is commonly used in veterinary practice on large animals because of its maggoticidal, antimicrobial and counter-irritating properties. As a counter irritant, it mediates acute inflammatory response. Heat is produced due to inflammation which helps in the dilatation of the blood vessels and increased blood flow [25], resulting in vascular permeability and infiltration of leukocytes followed by granuloma formation [26]. The macrophages and neutrophils are primary cells infiltrated locally at the site of inflammation. These cells cause clearance of necrotic tissue, blood clots and cellular debris which are essential for neovascularization, normal collagen deposition, healing and remodelling of the injured tissues [27,28].

Aflatoxicosis is often associated with clinic-pathological changes such as increases in total RBC count, PCV, haemoglobin and serum bilirubin concentration [29], which was also evident in our case from the haematological and biochemical profile of affected buffaloes. The hyperbilirubinemia observed in aflatoxicosis is often associated with the concentration dependent swelling and haemolysis [30]. The increased hepatocellular necrosis is known to increase concentration of both conjugated as well as unconjugated bilirubin in the serum [31], which corroborates our observations in the serum biochemical profile of aflatoxin-affected herd.

Aflatoxin has been reported to impair oocytes and the preimplantation development of embryos by overproducing reactive oxygen species (ROS) [32]. Penagos-Tabares et al. [33] reported a 75% abortion rate and an 18.8% decrease in the milk yield due to an aflatoxicosis outbreak in a dairy herd in Pakistan. In the present study, the effect on conception rate was evident and significantly affected the signs of oestrus due to which a limited artificial insemination (AI) could be performed. We could observe an increase of 91.66% in the abortion rate in the herd which was higher than the reported abortion rate [33]. Nutritional and therapeutic management responded well to the affected animals and the replacement of contaminated feeds with fresh ones brought the animals of the whole herd in activity, as well as normal feed consumption [34,35].

## 4. Conclusions

Aflatoxin contamination of feeds is a major problem especially in developing countries due to climatic variability and unorganised management in feed production. Routine check-ups of feeds are utmost importance to prevent the feeding of aflatoxin-contaminated feeds to the animals. Systemic efforts viz. therapeutic management with topical medicines, broad-spectrum antibiotics, supportive therapies with vitamins and antioxidants along with the replacement of contaminated feeds and inclusion of peta-sulphate mixture, and a toxin binder, are effective in the prevention and control of aflatoxicosis in buffaloes.

## 5. Materials and Methods

Central Institute for Research on Buffaloes (CIRB) is a research organisation under the aegis of the Indian Council of Agricultural Research (ICAR), Ministry of Agriculture and Farmers’ Welfare, Government of India. The institute has an animal farm of world-famous Indian riverine buffalo of Murrah breed at its Hisar campus for research and teaching activities. The animals on the farm are well maintained with uniform feeding and managemental practices.

### 5.1. Samples and Data

In the Animal Farm of ICAR-CIRB, Hisar (29.18° N and 75.70° E), India, some Murrah buffaloes (20 in no) were primarily found to be affected with tail gangrene in the age group of 1–3 years; hence, for the biochemical and clinical case study, the affected individuals were considered test subjects. To study the possible effect of the feed toxicosis outbreak, overall herd performances were assessed, where all the animals demonstrated feed refusal, sluggish movement, gastrointestinal dysfunction, reduced conception and milk production. The Animal Farm of ICAR-CIRB had a herd strength of 524 buffaloes comprising 136 buffaloes in milk, 31 dry buffaloes, 168 heifers, 69 bulls and 120 suckling calves in March 2023 when the outbreak was onset.

### 5.2. Clinical Investigation of Animals

**Figure 3:**
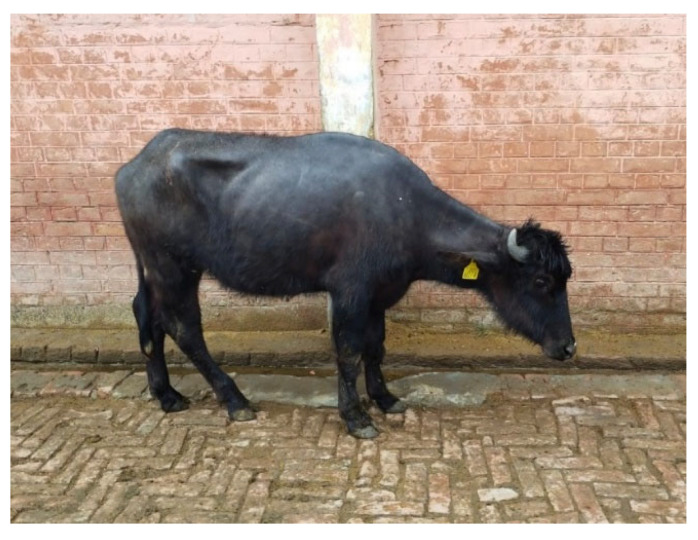
Aflatoxicosis-affected emaciated heifer.

**Figure 4:**
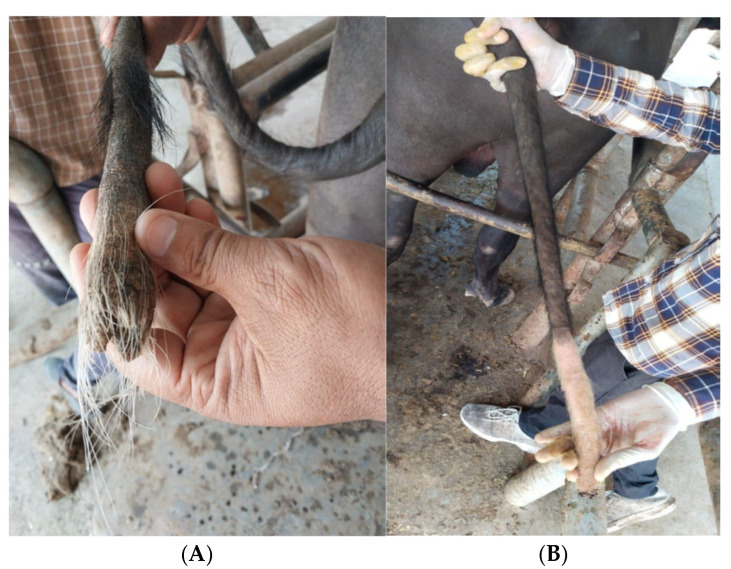
Tail gangrene and necrotic ring in affected animals (**A**,**B**).

The clinical signs and symptoms of the disease were recorded. There was no rise in body temperature with a reduced appetite of the animals. The body condition of all animals was deteriorating gradually showing progressive emaciation (Figure 3), lethargic condition and alopecia of the whole body. The tail of the animals was severely affected with gangrene (Figure 4) and thoroughly examined to delineate the length of tail necrosis, hair losses, oedematous and painful area and necrotic ring formation (Figure 4). The affected area of the tail was cold to touch due to the restricted blood supply. The tails were hyper-keratinised and looked scaly, leathery and greasy in appearance. After a thorough examination of the signs and symptoms of the disease, it was speculated that the tail gangrene may have been due to the presence of mycotoxins in the concentrate feeds offered to the animals. The severely affected buffaloes that died were subjected to post-mortem examination.

### 5.3. Clinical Investigation of Feed

To investigate the possible presence of mycotoxins in the feed ingredients, as suspected from the symptoms, feed ingredients as well as compound feed mixtures fed to farm animals were collected as per standard protocol and submitted to the Animal Feed Analytical and Quality Assurance Laboratory, Tamil Nadu Veterinary and Animal Science University, Namakkal, India, as per standard protocol for mycotoxin analysis.

### 5.4. Blood Collection of the Affected Buffaloes

Complete haematological and serum biochemical profiles of the affected individuals were carried out at the Department of Veterinary Clinical Complex Laboratory, LUVAS, Hisar. The blood samples were collected from jugular vein puncture by a trained veterinarian of the farm, as per the standard protocol.

### 5.5. Investigation of Production and Reproduction Parameters

To investigate the health effects on the overall production and reproduction performance of the farm buffaloes, the cumulative herd data about the month-wise conception rate, wet and herd averages were compared against the average of the past two years’ month-wise performance records for 7 months (March to September). A chi-square goodness of fit was tested assuming the null hypothesis that there is no significant difference between the performance observed in 2023 and the performance expected as per the past 2 years’ performance. The critical values of chi-square were obtained at α = 0.05 and 6 degrees of freedom.

### 5.6. Therapeutic Interventions for Affected Animals

The hairs around the affected area of the tail were slipped and the tail was washed with a diluted (0.01%) solution of KMNO_4_. The greasy or oily exudate deposited on affected parts was cleaned with methylated spirit. After drying, the dressing was conducted with povidone iodine solution (10%) and repeated twice a day for two consecutive days. Phenyl (Bengal Chemicals and Pharmaceuticals, Kolkata, India) in diluted form at various concentrations [on days 3–4 (1:4), 5–6 (1:2) and 7–8 (1:1)] was used twice a day for dressing the affected tail region. The bandages wrapped around the affected part of the tail were replaced on alternate days. After day eight, the tail dressing was continued with Lorexane^®^ ointment (Virbac Animal Health Pvt. Ltd., Mumbai, India), and Betadine^®^ (Win-Medicare Pvt. Ltd., New Delhi, India) for the next week. Systematically, an oxytetracycline long-acting (Bioxy-LA^®^, Brilliant Bio-pharma, Hyderabad, India) of 10 mg per kg body weight with repetition on days 0, 3 and 6 and meloxicam (Melonex^®^, Intas Pharmaceuticals Ltd., Ahmedabad, India) of 10 mg per kg body weight daily for 7 days were injected intramuscularly to the affected buffaloes. An injection (S/c) ivermectin of 1 mL per kg body weight (two times at weekly intervals) and an injection (I/m) L-carnitine (Dozliv^®^, Carus Laboratories Pvt. Ltd., Karnal, India) of 10 mL per animal for 10 days; a multivitamin injection (V-5^®^, Care Vet Pharma, Ludhiana, India) of 5 mL I/m at weekly intervals for four shots and a multimineral injection, Stimvet^®^ (Wellcon Animal Health Pvt. Ltd., Agra, India) of 5 mL per animal once only were used.

### 5.7. Nutritional Supplements

To eliminate the possible presence of causative agents in the feeds, all feed constituents (wheat straw, green fodder and concentrate mixtures) were immediately replaced with fresh ones. A toxin binder (HSCAS @ 1.5 kg/ton) was added to the concentrate mixture of all the animals. Additionally, a penta-sulphate mixture (ferrous sulphate, 166 g; copper sulphate, 24 g; zinc sulphate, 75 gm; cobalt sulphate, 15 g; and magnesium sulphate, 100 g) was prepared at the laboratory and supplemented at the dose rate of 30 g daily per animal by mixing with concentrate mixture for 20 days.

## Figures and Tables

**Table 1:** Description of tail gangrene in aflatoxin-affected buffaloes.

Tails Conditions	Length of Tail Affected (Inches)			
	<3 Inches	3–6 Inches	6–12 Inches	>12 Inches
Tail alopecia	5	8	3	4
Tail necrosis	6	10	2	2
Oedematous and painful tail	2	9	5	4
Necrotic rings	1	4	1	0

**Table 2:** Age-wise clinical history and post-mortem findings of affected buffaloes.

Month	No. of Animals Died	Age Group (Months)	Clinical History	Post-Mortem Findings
March	4	0–3	Ataxia, hypothermia, laboured breathing, circular movement	Hepatitis and nephritis
	1	3–6	Ataxia, hypothermia, laboured breathing, circular movement	Hepatitis and nephritis
April	1	3–6	Pot-belly, hypothermia, dyspnoea, circular movement and ataxia	Liver cirrhosis, haemorrhagic enteritis, ulcerative abomasitis, nephritis
	4	6–12	Pot-belly, hypothermia, dyspnoea, circular movement and, froth, shooting bubbly diarrhoea, recurrent tympany, chronic debility and tail gangrene	Liver cirrhosis, haemorrhagic enteritis, ulcerative abomasitis, nephritis
May	13	6–12	Pot-belly, hypothermia, dyspnoea, circular movement and, froth, shooting bubbly diarrhoea, recurrent tympany, chronic debility and tail gangrene	Liver cirrhosis, haemorrhagic enteritis, ulcerative abomasitis, nephritis
June	1	6–12	Pot-belly, hypothermia, dyspnoea, circular movement and, froth, shooting bubbly diarrhoea, recurrent tympany, chronic debility and tail gangrene	Liver cirrhosis, haemorrhagic enteritis, ulcerative abomasitis, nephritis
	3	12–24	Pot-belly, hypothermia, dyspnoea, circular movement and, froth, shooting bubbly diarrhoea, recurrent tympany, chronic debility and tail gangrene	Liver cirrhosis, haemorrhagic enteritis, ulcerative abomasitis, nephritis
July	2	12–24	Progressive emaciation, dyspnoea, diarrhoea, normothermia, chronic debility, ascites and tail gangrene	Liver cirrhosis, haemorrhagic enteritis, ulcerative abomasitis, nephritis
	2	>24	Progressive emaciation, dyspnoea, diarrhoea, normothermia, chronic debility, ascites and tail gangrene	Liver cirrhosis, haemorrhagic enteritis, ulcerative abomasitis, nephritis
Aug	2	>24	Progressive emaciation, dyspnoea, diarrhoea, normothermia, chronic debility, ascites and tail gangrene	Multiple organ failure and coccidiosis
Sept	5	>24	Ascites and laboured breathing	Hydrothorax, hepatitis, peritonitis and pneumo-enteritis

**Table 3:** Aflatoxin level in different feed ingredients and concentrate mixtures fed to experimental buffaloes.

S. No.	Feed Ingredients	Aflatoxin Level (ppb)	
		B-1	B-2
1	Maize grain	722	52
2	Wheat grain	10	nd
3	Oats grain	nd	nd
4	Barley grain	nd	nd
5	Groundnut cake	4695	586
6	Cottonseed cake	939	59
7	Mustard cake	62	10
8	Wheat bran	nd	nd
9	Guar korma	nd	nd
10	Concentrate feed mixture	822	88

nd = Not detected.

**Table 4:** Comparative monthly record of animal performances during pre- and post-exposure of toxins.

	AI Performed (No.)		Conception Rate (%)		Milk Yield (kg/d)			
					Wet Average		Herd Average	
Months	2021–2022	2023	2021–2022	2023	2021–2022	2023	2021–2022	2023
March	33	26	49.37	42.31	9.95	9.99	7.53	8.06
April	21	20	33.07	40.91	9.73	9.44	7.16	7.33
May	26	22	49.77	40.91	9.83	9.02	7.00	6.39
June	24	9	35.57	11.11	9.82	9.32	6.86	6.31
July	26	9	42.26	11.11	10.16	9.51	6.95	5.90
August	36	32	40.02	56.25	9.80	9.80	6.65	6.00
September	50	46	50.16	56.25	10.05	10.18	6.93	6.47
Mean	30.86	23.43	42.89	36.98	9.91	9.61	7.01	6.64
SEM	3.15		3.75		0.088		0.159	
Significance	0.579		0.046		0.025		0.036	

## Data Availability

The original contributions presented in this study are included in the article/supplementary material. Further inquiries can be directed to the corresponding authors.

## Supplementary Materials

The following supporting information can be downloaded at https://www.mdpi.com/article/10.3390/toxins17020097/s1. Table S1: Common gross and histopathological findings in the vital organs of the carcasses; Table S2: Haematological findings of blood samples collected from affected buffaloes; Table S3: Serum biochemical profile of affected buffaloes.
