# Supplementary material for: Delineation of Aflatoxicosis on Health and Performance of Water Buffalo (Bubalus bubalis) and Its Therapeutic and Nutritional Management
Source: Toxins (Basel). 2025 Feb 18;17(2):97. doi: 10.3390/toxins17020097 (PMC11860594; doi:10.3390/toxins17020097)
Supplement: Supplementary file 1 [file toxins-17-00097-s001.zip › toxins-3430301-supplementary.pdf]

Supplementary Materials: Delineation of Aflatoxicosis on Health and Performance of Water Buffalo (*Bubalus bubalis*) and Its Therapeutic and Nutritional Management

Rajesh Kumar, Sanjay Kumar, Supriya Chhotaray, Madhu Singh, Rupali Rautela and Avijit Dey

Table S1. Common gross and histopathological findings in the vital organs of the carcasses.

S. No.	Organ	Gross findings	Histopathological findings
1.	Abomasum	Mucosal folds thick and edematous, mucosa congested, haemorrhagic and ulcerative	Abomasitis, necrosis, infiltration of MNCs, congestion and haemorrhages
2.	Intestines	Small and large intestines revealed mucosal thickening, congestion, marked ecchymotic hemorrhages	Necrosis, denudation of mucosal epithelium, severe infiltration of MNCs, congestion and haemorrhages
3.	Lungs	Congested and oedematous	Serous pneumonia along with alveoli filled with serous fluid, scattered mononuclear cells and polynuclear cells, severe congestion and haemorrhages
4.	Liver	Pale and white discoloration, hard in consistency, Cirrhosis, gall bladder distended	Diffused fatty changes, congestion, cirrhosis
5.	Spleen	Petechial haemorrhages	Necrosis and depletion of the lymphocytes in the white pulp and focal haemorrhages
6.	Heart	Hydro-pericardium, petechial to ecchymotic haemorrhages	Pericarditis, infiltration of MNCs along with severe hemorrhages in pericardium and myocardium
7.	Kidneys	Pale yellow discoloration	Thickening of the capsule, congestion and haemorrhages in peritubular capillaries
8.	Brain	Congestion and haemorrhages in focal area	Severe congestion and hemorrhages in focal area, edema, gliosis, neuronal degeneration and satellitosis in focal area

Table S2. Haematological findings of blood samples collected from affected buffaloes.

Haematological Parameters	B1	B2	B3	B4	B5	B6	B7	B8	B9	B10	B11	B12	B13	B14	B15	B16	B17	B18	B19
Hb (g/dl)	12.7	9.7	12	9.3	7.9	12.2	12.3	12.5	12.3	12.3	10.5	11.1	11.4	11.8	10.1	8.5	11.7	11.3	8.3
TEC (Million/cumm)	8.58	5.74	8.64	5.90	5.26	8.48	8.20	8.10	7.42	7.87	6.37	5.96	6.69	8.20	6.42	5.76	7.35	7.57	5.82
TEC (Thousand/cumm)	15.89	10.68	17.62	14.16	18.12	27.78	16.28	9.26	35.89	12.43	12.11	7.42	12.32	16.43	9.24	11.35	16.59	9.01	9.17
PCV (%)	44	28	43	32	28	45	44	44	41	40	40	44	44	44	30	30	43	46	32
MCV (F1)	51.6	49.5	53.3	53.9	54.1	53	53.8	54.4	54.8	51.3	62.8	73.4	65.3	53.9	46	52	58.6	60.2	55.4
THR (Thousand/cumm)	138	947	158	181	122	183	176	193	500	220	171	153	111	190	265	259	182	181	141
Lymphocytes (%)	68	61	56	49	54	29	51	67	23	51	50	38	51	42	59	53	60	54	49
Monocytes (%)	1	5	1	1	1	1	2	1	2	1	1	1	1	2	3	2	1	2	2
Granulocytes (%)	31	34	43	50	45	70	47	32	75	48	49	61	48	56	38	45	39	44	49
Haemoprotozoan parasite	-Ve	-Ve	-Ve	-Ve	-Ve	-Ve	-Ve	-Ve	-Ve	-Ve	-Ve	-Ve	-Ve	-Ve	-Ve	-Ve	-Ve	-Ve	-Ve

Note: B1-B19 indicates different buffaloes affected due to aflatoxicosis

Table S3. Serum biochemical profile of affected buffaloes.

Animals No.	ALP	GGT	SGOT	SGPT	Direct bilirubin	Total bilirubin	Total Proteins	Albumin	Glucose	Total calcium	Phosphorus	Magnesium	Urea	Creatinine
B1	565	100.8	229.6	20.2	3.01	10.93	5.4	1.38	12.1	7.9	4.77	2.39	22.9	0.08
B2	599	57.6	166.7	14.1	0.24	4.12	4.33	1.66	31	7.8	5.81	1.74	21.8	0.25
B3	198	30.5	185.5	27.2	0.4	0.85	6.29	1.66	46.2	9	7.54	3.06	51.6	1.92
B4	753	191.4	138.7	38.4	0.08	0.1	4.25	1.97	23.2	10.2	7.06	1.48	28	0.72
B5	684	117.8	196.2	26.9	0.35	2.41	6.3	1.48	19.9	7.5	6.34	2.55	16.3	0.14
B6	212	14.9	165.5	19.6	0.01	0.09	5.85	2.51	56.8	12	8.32	2.37	17.1	1.21
B7	218	52.4	98.7	46	0.1	0.13	4.66	2.19	31.6	9.9	5.48	1.74	19.6	1.03
B8	202	44.6	157.7	43.6	0.08	0.08	4.33	2	16.6	9.3	8.29	1.71	17.1	0.64
B9	338	136.5	176.6	41.1	0.34	0.76	7.17	2.28	24.5	9.4	3.67	2.88	28.6	0.77
B10	488	28.4	154.2	46.3	0.24	1.66	4.93	1.99	19	9.5	4.96	2.09	12.2	0.22
B11	640	NA	184.3	25	0.37	2.74	5.79	1.16	25.9	8.7	3.61	3.29	55	0.59
B12	123	43.3	102.3	41.7	0.08	0.08	6.71	2.24	18.8	9.6	5.94	2.55	24.8	1.16
B13	475	77.8	257.4	22.6	0.29	2.64	6.25	1.29	16.2	7.1	4.28	2.47	35.5	0.48
B14	1080	49.8	224.3	31.4	0.51	5.09	6.76	1.22	40.4	9.1	5.22	2.65	28.6	0.59
B15	NA	59.9	246.9	22	1.29	7.9	7.16	1.51	18.3	8.6	3.49	2.54	35.2	0.08
B16	1080	41.2	130.9	37.8	0.31	0.73	5.94	1.75	35.3	9.6	6.08	2.65	11.5	0.66
B17	300	25.4	199.8	19.9	0.11	0.12	5.44	2.31	69.1	10.5	6.81	1.98	12.6	1.11
B18	399	46.3	109.8	17.8	1.21	6.36	4.82	1.18	19.8	7.6	4.49	1.59	16.3	0.61

Note: B1-B18 indicates different buffaloes affected due to aflatoxicosis
